# Pennsylvania

**Published:** 1897-01

**Authors:** 


### CONTROL WORK.

*Pennsylvania.* Up to November 25th there had been examination applications to the State Veterinary Sanitary Board for 3226 head of cattle. Of this number, 2975 had been examined with tuberculin, of which 795 were condemned and destroyed as tuberculous at a total cost for the entire work of $18,229, or an average of less than $23 per head.

The Food Commissioner of Pennsylvania in his annual report states that there is an unusual demand for the literature of the Board relative to the adulteration of milk by the use of so-called preservatives. The rapid increase of creameries in the State has presented two new and important aspects of the milk question. First, the production and sale of a very poor quality of skim-milk for feeding purposes; second, the danger of spreading tuberculosis when feeding this to young animals, if there should be any diseased herds within the circuit of the factory's patrons. He reports 1,000,000 milch cows in the State, producing 1,700,000,000 quarts of milk. Of this amount at least 600,000,000 quarts are consumed as milk and cream. The pure-food law has proved of more value in dealing with the milk-question than if it set up a legal standard. He well says that if the latter is low enough to be fair to the dairyman at different seasons of the year, and for all breeds of cattle, the temptation is offered to the dealer having milk rich in fat to adulterate the milk, knowing he could do so and still keep within the standard fixed as legal. He advises consumers of milk to decline to receive the milk unless proper assurance is given, backed by a report from some competent veterinarian, that the cows furnishing the same are healthy.

Twenty-one cattle in a Wayne County herd were recently condemned and destroyed as tuberculous.

